# YTHDC1 positively regulates PTEN expression and plays a critical role in cisplatin resistance of bladder cancer

**DOI:** 10.1111/cpr.13404

**Published:** 2023-04-17

**Authors:** Yinjie Su, Bo Wang, Jian Huang, Ming Huang, Tianxin Lin

**Affiliations:** ^1^ Department of Urology, Sun Yat‐sen Memorial Hospital Sun Yat‐sen University Guangzhou China

## Abstract

Activation of PI3K/AKT signalling by PTEN loss significantly enhances chemoresistance in bladder cancer. This study aims to evaluate PTEN regulation and identify targets that could be used to relieve chemoresistance. Expression of YTHDC1, γ‐H2AX and PTEN were detected by IHC assay. Cell Counting Kit‐8 assay, colony formation assay and tumour xenograft experiment evaluated cisplatin response. Flow cytometry and comet assay estimated cell apoptosis, cell cycle distribution and DNA repair capability. Quantitative real‐time polymerase chain reaction, Western blot and RIP assay assessed binding properties between PTEN mRNA and YTHDC1. Silencing YTHDC1 in bladder cancer cells reduced PTEN expression and activated PI3K/AKT signalling by destabilizing PTEN mRNA in an m^6^A‐dependent manner. Low YTHDC1 expression indicated poor cisplatin sensitivity in bladder cancer patients. Reducing YTHDC1 expression promoted drug resistance to cisplatin, while over‐expressing YTHDC1 promoted cisplatin sensitivity. Reducing YTHDC1 expression activated DNA damage response, which includes quicker cell cycle recovery, apoptosis evasion and an enhanced DNA repair capability, whereas these effects were attenuated when MK2206, a PI3K/AKT inhibitor was applied. We provide novel evidence that PTEN/PI3K/AKT signalling pathway could be regulated by YTHDC1 in an m^6^A‐dependent manner and highlight a critical role of YTHDC1 in cisplatin resistance of bladder cancer.

## Graphical abstract

As illustrated, lower YTHDC1 expressed cancer cells indicate poor cisplatin outcome in bladder cancer patients; lower YTHDC1 destabilizes PTEN mRNA, thus activates AKT‐associated DNA damage response and attenuates cisplatin‐induced DNA damage.

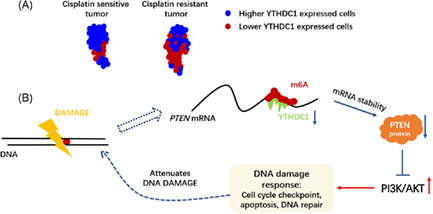

## 1 INTRODUCTION

Although chemotherapy effectively mitigates pathologic progression in advanced bladder cancer patients, the response rate remains not satisfactory. There are around 50% patients show intrinsic‐ or acquired‐ drug resistance.^1^, ^2^ Cisplatin is frequently used in bladder cancer chemotherapy,^1^, ^2^, ^3^ however, it induces DNA damage and leads to toxicity which could contribute to the cell death.^3^, ^4^ Cisplatin resistance is induced when tumour cells enhance DNA repair capacity, evade apoptosis, trigger autophagy and drug efflux by activation of oncogenes or suppression of tumour suppressors.^5^, ^6^

Among multiple genes that altered and lead to chemoresistance, PTEN plays an ultimate role.^7^ Loss of PTEN expression has been detected in a wide range of human cancers.^8^ Absence of PTEN function contributes to constitutive activation of PI3K/AKT signalling,^9^ thus promoting evasion of cisplatin‐induced cell apoptosis and drug resistance.^10^, ^11^, ^12^ Considering the immense influence of PTEN on cells, it is still unclear how PTEN is downregulated in cancer. Hence, further research on how PTEN is regulated would help to identify targets that could be used to relieve chemoresistance and tumour progression.

Regulations that affect PTEN expression vary from gene copy number regulation to post‐transcriptional modification.^8^ *N* ^6^‐Methyladenosine (m^6^A) is one of the common ways of RNA modification which is responsible for modulating the stability, splicing and translation of modified mRNAs.^13^, ^14^ Recent studies have suggested that enhanced activity of m^6^A methyltransferase generally promotes malignancy in bladder cancer,^15^, ^16^, ^17^, ^18^ and m^6^A modification on PTEN mRNA oppositely leads to stabilized PTEN expression.^19^, ^20^ Given the tumour suppressive role of PTEN in cancers,^9^ there should be a mechanism that supports PTEN loss in bladder cancer from the view of m^6^A modification.

A complete event of m^6^A modification is performed by cooperation of three classes of proteins.^13^ Except for the installation of ‘writer’ and m^6^A clearance by ‘eraser’, ‘reader’ recognizes these marked sites and is responsible for determining the consequences of an m^6^A modified RNA.^13^, ^14^ Facing the enhanced PTEN expression induced by m^6^A modification in cancers,^19^ expression of YTHDC1 (YTH domain containing 1), one of the ‘readers’, discriminately destabilized PTEN mRNA which was discovered to mediate neuronal survival and ischemic stroke.^21^ By analysing TCGA bladder cancer dataset, it was found that *YTHDC1* expresses at low level in tumour cells, especially in advanced tumour types. Lower expression of *YTHDC1* showed a significant correlation with lower *PTEN* expression. These findings suggest that YTHDC1 might be responsible for PTEN loss in bladder cancer and play a critical role in cisplatin resistance.

Hence, we next compared expression of YTHDC1 in a cohort of bladder cancer patients. We found that lower expression of YTHDC1 indicated less cisplatin sensitivity, DNA damage degree and PTEN expression. Based on the findings above, we constructed stably YTHDC1‐silenced bladder cancer cells. We found that silencing YTHDC1 affected DNA damage response (DDR) and promoted cell survival via destabilizing PTEN mRNA in an m^6^A‐dependent manner, and these effects were attenuated when MK2206, a PI3K/AKT inhibitor was applied. This study demonstrates that YTHDC1 in bladder cancer is a critical factor for cisplatin resistance. Moreover, we uncover a novel epi‐transcriptomic mechanism to explain PTEN loss in bladder cancer.

## 2 MATERIALS AND METHODS

### 2.1 Cell culture

SV‐HUC‐1 and a panel of human bladder cancer cells, including T24, UM‐UC‐3 (U3), 5637, RT4 and J82 cell lines, were purchased from ATCC. All cells were cultured in complete DMEM medium except for SV‐HUC‐1 cell, which was cultured in complete DMEM/F12 medium. Each medium was routinely supplemented with 10% fetal bovine serum (FBS), 1% penicillin and streptomycin antibiotics and 1% mycoplasma removal reagent. Cells were cultured in an atmosphere of 37°C with 5% CO_2_.

### 2.2 Chemicals and antibodies

Cisplatin (*cis*‐diamminedichloroplatinum II), Etoposide (VP‐16) and MK2206 were purchased from Selleck. Puromycin sulphate was purchased from Beyotime Biotechnology. Anti‐YTHDC1 (CST and Abcam), anti‐m^6^A (Abcam), anti‐PTEN (Proteintech), anti‐phosphorylated AKT (ser473) (CST), anti‐γH2AX (ser139) (CST) and anti‐GAPDH (Beyotime Biotechnology) primary antibodies were used in this study. Goat anti‐mouse and rabbit IgG‐HRP (Abcam) were used as secondary antibodies for Western blots. The secondary antibody for immunohistochemistry (IHC) was obtained from a SP Rabbit & Mouse HRP Kit (Cwbiotech).

### 2.3 IHC staining and evaluation

Tissue slides were collected from Sun Yat‐sen Memorial Hospital and Sun Yat‐sen University Cancer Center. All samples were fixed in formalin and embedded in paraffin. After dewaxing with dimethylbenzene and dehydration in gradient ethanol, sections were quickly immersed in 3% hydrogen peroxide at room temperature for 10 min to quench endogenous peroxidase activity, and then incubated in Tris‐EDTA buffer (pH 9.0) at 100°C for 30 min for antigen retrieval. Sections were incubated with the primary antibody (anti‐YTHDC1, 1:1000; anti‐PTEN, 1:2000; anti‐γ‐H2AX, 1:400) overnight at 4°C. After washing with phosphate‐buffered saline (PBS), the sections were incubated with secondary antibody at room temperature for 30 min and stained with DAB and haematoxylin. Positive cells presented apparent brown staining. Sections were photographed under a microscope in five random fields. Protein level was scored according to the multiplication of staining intensity (0 = no staining, 1 = weak staining, 2 = moderate staining, 3 = strong staining, 4 = very strong staining) and percentage of stained cells (0 = 0%, 1 ≤ 10%, 2 ≤ 20%, 3 ≤ 30%, …, 9 ≤ 90%, 10 ≤ 100%).

### 2.4 RNA extraction and quantitative real‐time polymerase chain reaction

Cell samples were processed directly with RNAiso Plus (Takara) according to manufacturer's protocol for RNA extraction. Extracted RNA was reversely transcribed to cDNA via using a RRO36A kit (Takara). The primers used were: YTHDC1: F‐TCAGGAGTTCGCCGAGATGTGT, R‐AGGATGGTGTGGAGGTTGTTCC; PTEN: F‐TGAGTTCCCTCAGCCGTTACCT, R‐GAGGTTTCCTCTGGTCCTGGTA; GAPDH: F‐GTCTCCTCTGACTTCAACAGCG, R‐ACCACCCTGTTGCTGTAGCCAA. A total of 10 ng cDNA was applied for quantitative real‐time polymerase chain reaction (PCR) with a RR820A kit (Takara). Cycle threshold values were determined and the correlated fold change was analysed.

### 2.5 Protein extraction and Western blot

Cell samples were collected and quickly incubated with cold RIPA‐strong lysis buffer (Beyotime Biotechnology) which contains 1% protease inhibitor cocktail (Cwbiotech) and 1% phosphatase inhibitor (Cwbiotech) over 30 min. The lysis sample was then centrifuged at 12,500 rpm at 4°C for 30 min. The supernatant was collected, and a BCA (Thermo Fisher Scientific) assay was used for protein quantitation. A total of 30 μg of protein sample was added to each lane of a sodium dodecyl sulphate–polyacrylamide gel. Proteins were transferred to a polyvinylidene fluoride membrane (0.2 μm; Millipore) after electrophoresis. The membrane was then blocked with 5% skimmed milk at room temperature for 1 h and incubated with the indicated primary antibodies overnight at 4°C. All primary antibodies used in this research were diluted in 1:1000. Later, the membranes were washed with PBST buffer and incubated with secondary antibody for 2 h at room temperature. The secondary antibodies were diluted in 1:10,000. Finally, the targeted protein band was illuminated with ECL Western Blotting Substrate (Millipore).

### 2.6 Construction of stably YTHDC1 silenced/over‐expressed cells

Lenti‐viruses that express shRNAs for YTHDC1 were purchased from Shanghai Genechem. The lenti‐viruses were constructed with hU6‐MCS‐Ubiquitin‐EGFP‐IRES‐puromycin plasmid that expressing sequence of transcribed shRNAs, which were as follows: sh‐YTHDC1‐1: 5′‐TGGATTTGCAGGCGTGAAT‐3′; sh‐YTHDC1‐2: 5′‐GCGAGATAGAGGACGTGAT‐3′. The sh‐YTHDC1/NC viruses were used to infect T24 and U3 cells separately according to manufacturer's instructions. Lenti‐virus that over‐expressing YTHDC1 was purchased from Guangzhou IGE Biotechnology. Lenti‐virus was constructed with pCDH‐CMV‐Homo‐YTHDC1‐EF1‐copGFP‐T2A‐Puromycin plasmid. The YTHDC1/Vector viruses were used to infect T24 cells separately according to manufacturer's instructions. Puromycin was used to select a colony that stably expressed the plasmid. The knockdown/over‐expression efficiency were evaluated by quantitative real‐time PCR and Western blot.

### 2.7 Cell Counting Kit‐8 assay

Approximately 1000 cells were seeded in each well of a 96‐well plate (Corning). After 24 h, a series dose of cisplatin (0, 1, 5, 10, 20, 40, 80, 100 μM) was added. Cells were first treated with cisplatin for 4 h. Then, the medium was changed to complete medium for another 48 h of culture. The cells were then incubated with medium containing 10% Cell Counting Kit‐8 buffer (Beyotime Biotechnology) for 2 h at 37°C, and the indicated OD value was detected at 450 nm.

### 2.8 Colony formation assay

Approximately 1000 cells were seeded in a six‐well plate (Corning). After 24 h, cisplatin with 20 μM was added. After 4 h of treatment, medium that contains cisplatin was replaced with complete medium and cultured for another 2 weeks. The cells were fixed with 4% paraformaldehyde and stained with 1% crystal violet. Colonies that contain over 50 cells were counted as an individual.

### 2.9 Tumour xenograft experiment

Approximately 1 × 10^6^ T24 bladder cancer cells were subcutaneously injected into the flanks of 4‐week‐old female BALB/c nude mice. After tumour was formed, cisplatin (3 mg/kg) was given to all mice through intra‐peritoneal injection, which was performed once a week. Length (*L*) and width (*W*) of tumour were measured every 3 days. Tumour size was calculated according to the formula *V* = *L* × *W* ^2^/2. At the end of experiment, all mice were sacrificed by cervical dislocation and the tumours were dissected.

### 2.10 Cell cycle analysis

Cells were trypsinized into a single‐cell suspension, washed with cold PBS, fixed in 70% cold ethanol and stored at −20°C. Samples were centrifuged and washed with PBS before analysis. 500 μl of PBS and 10 μl of DNase‐free RNase (10 μg/ml) were added and incubated with the cells for 30 min at 37°C, and then, 150 μl of propidium iodide (50 μg/ml) was added and incubated with the cells for 10 min at room temperature in the dark. Cell aggregates were filtered, and DNA content was detected by flow cytometry. Modfit software was used to analyse the results.

### 2.11 Apoptosis analysis

Cells were trypsinized, washed with cold PBS and gently pipetted into a single‐cell suspension with 100 μl of cold PBS. An Annexin V‐AF647/PI kit (ESscience) was used for cell labelling. The procedures in this experiment were performed according to manufacturer's protocol. Apoptotic cells were detected by flow cytometry, and cytoExpert software was used to analyse the results. The total apoptotic rate includes the sum of early and late apoptotic cells.

### 2.12 Immunofluorescence assay

Cells were seeded into a confocal dish (diameter of 15 mm) for 24 h, and then treated with cisplatin (20 μM) or etoposide(10 μM). After 48 h of treatment, the cells were fixed with 4% paraformaldehyde for 10 min and permeabilized with 0.2% Triton X‐100 in PBS for 15 min at room temperature. Then, the cells were blocked with 3% BSA containing 1% FBS for 1 h, and incubated with the indicated primary antibodies overnight at 4°C. All primary antibodies used in this assay were diluted with 1:400 in PBS with 1% BSA. Later, the cells were washed with PBS and incubated with secondary antibody (Alexa Fluor 488 for Rabbit and Alexa Fluor 594 for mouse; Proteintech) for 1 h in the dark at room temperature. The secondary antibodies were diluted 1:200 in PBS with 1% BSA. After counterstaining with DAPI (Servicebio), the cells were observed and photographed by using a confocal microscope (Olympus).

### 2.13 Alkaline comet assay for DNA repair assessment

Cells were trypsinized, washed with cold PBS and gently pipetted into a single‐cell suspension at a density of 10,000 cells/ml. A reagent kit for the single‐cell gel electrophoresis assay (catalogue # 4250‐050‐K; Trevigen) was used. All steps were executed according to manufacturer's protocol. Gel after electrophoresis was observed microscopically. CometScore 2.0 software was used to analyse the results. The DNA percentage in the tail and tail length were used as descriptors of DNA damage.

### 2.14 RNA‐binding protein immunoprecipitation

Cells were trypsinized and washed with cold PBS. A magna RIP kit (Catalogue No. 17‐700; Millipore) was used. All steps were executed according to manufacturer's protocol. RNA that bound with indicated protein was evaluated by quantitative real‐time PCR, and the fold change was analysed as compared with that was expressed in IgG dragging group.

### 2.15 Actinomycin D treatment for RNA stability assessment

Approximately 1 × 10^5^ cells were seeded in six‐well plate. Then, 5 μg/ml actinomycin D (CST) was added for 0, 2, 4, 8 and 12 h. Cells were collected for RNA extraction at different indicated times. A total of 500 ng of RNA was reversely transcribed for cDNA synthesis and quantitative real‐time PCR analysis.

### 2.16 Statistical analysis

Statistical analysis was conducted with Graphpad Prism 8.0 software. A *t*‐test was used to assess quantitative data between two groups. For overall survival analysis, the Kaplan–Meier method was applied, and a significant difference between groups was evaluated by using a log rank test. Correlations between protein expression levels were analysed by using minimum binomial regression. *p < 0.05 and **p < 0.01 were considered significant for all statistical tests.

## 3 RESULTS

### 3.1 YTHDC1 positively regulates PTEN expression in bladder cancer

**FIGURE 1:**
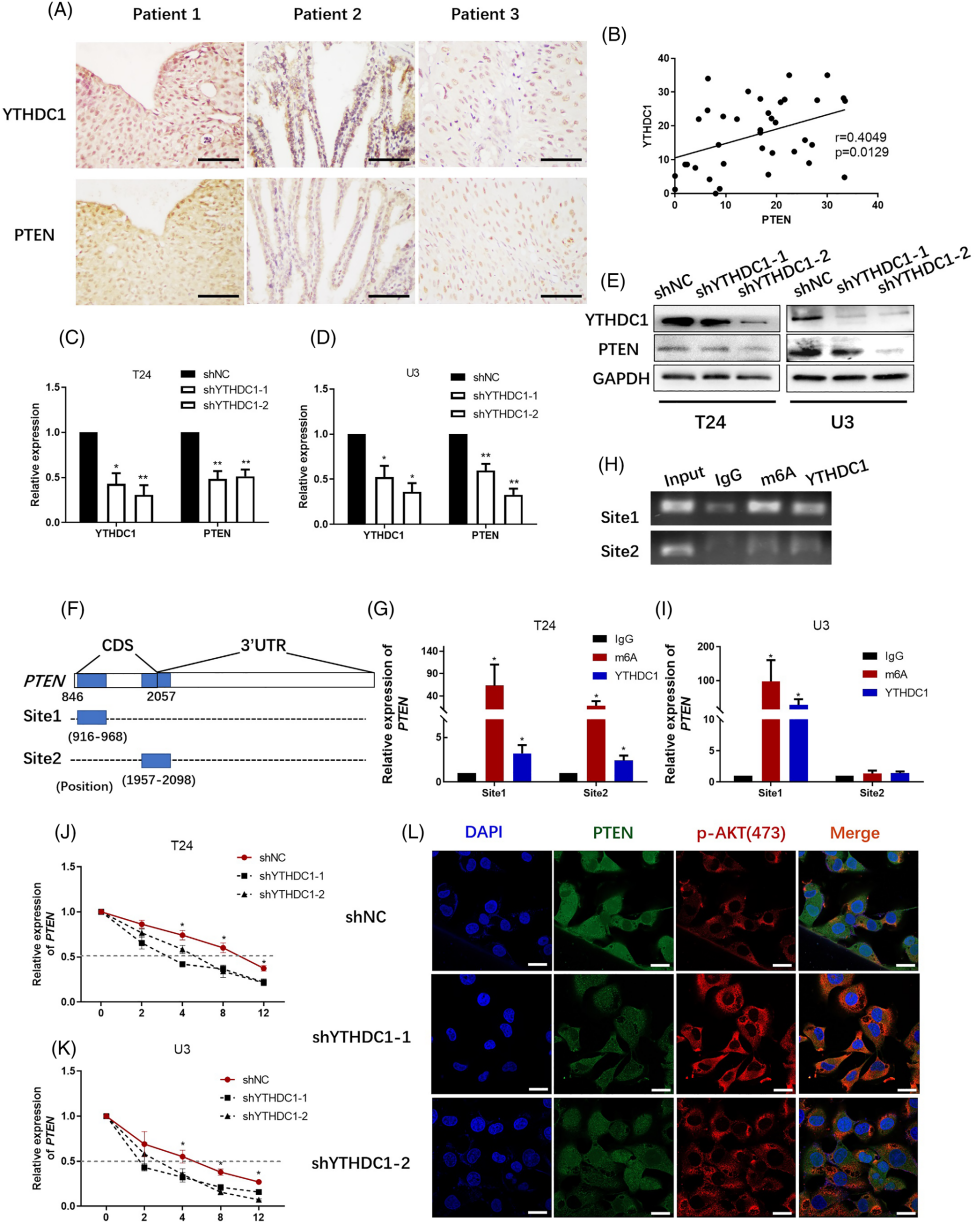
YTHDC1 positively regulates PTEN expression in bladder cancer. YTHDC1 and PTEN level were detected by an immunohistochemistry assay in tumour sections from 37 bladder cancer patients. The correlation between YTHDC1 and PTEN levels was analysed with Graphpad Prism 8.0 software. (A) Representative images show YTHDC1 and PTEN expression in three identical patients. The scale bar indicates 50 μm. (B) A scatter plot shows the correlation between YTHDC1 and PTEN levels. The expression of YTHDC1 and PTEN levels were analysed by (C and D) quantitative real‐time polymerase chain reaction (PCR) and (E) Western blot. Data are presented as the mean ± SEM, and experiments were performed at least three times. GAPDH was applied as an internal control. (F) Modification of m^6^A on PTEN mRNA was predicted by using SRAMP database (www.cuilab.cn/sramp). Schema illustrates the predicted mode. (G–I) The binding potential between YTHDC1 and PTEN mRNA was evaluated by using a RIP assay. IgG was taken as a negative control. (H) Representative images show pulldown products in T24 bladder cancer cells by performing agarose gel electrophoresis. Data are presented as the mean ± SEM, and experiments were performed at least three times. (J and K) The cells were treated with 5 μg/ml actinomycin D, and the expression of *PTEN* was detected by quantitative real‐time PCR. Data are presented as the mean ± SEM, and experiments were performed at least three times. (L) The expression of PTEN and p‐AKT(ser473) were evaluated via performing an immunofluorescence assay. T24 bladder cancer cells were captured microscopically and the scale bar indicates 50 μm. Experiments were performed at least three times.

We evaluated the expression of PTEN and YTHDC1 in a cohort of bladder cancer patient tissues we collected. It was found that the expression of PTEN shows a positive linear correlation with YTHDC1 level (Figure 1A,B). The relationship between these two factors was similarly confirmed by analysing TCGA bladder cancer datasets (Figure S1A). Besides, in bladder cancer cells, silencing YTHDC1 obviously reduced PTEN expression at both mRNA (Figure 1C,D) and protein (Figure 1E) levels. These results indicate a role of YTHDC1 in regulating PTEN expression in bladder cancer.

To identify how PTEN is regulated by YTHDC1, we predicted possible m^6^A binding sites on PTEN mRNA by using SRAMP database (www.cuilab.cn/sramp). Two potential binding sites were found and primers that enable completely covering the binding region were designed by using the www.primer3.com (Figure 1F). The binding capability between YTHDC1 and PTEN mRNA was evaluated with a RIP assay. In this experiment, compared with the IgG dragging group, mRNA of PTEN could be significantly enriched by both the m^6^A and YTHDC1 antibodies (Figure 1G,I). This result demonstrated a binding affinity between YTHDC1 and PTEN mRNA.

As an m^6^A reader, covalent mechanisms to regulate the gene expression and activity include alternative RNA splicing, mRNA degradation and cytoplasmic‐nuclear transportation.^14^ To identify the mechanism by which YTHDC1 regulates PTEN expression, we performed mRNA sequencing between shNC and shYTHDC1 cells. We did not find any significant differences in the alternative splicing form of *PTEN* between shNC and shYTHDC1 cells. Actinomycin D impairs global transcriptional activity and is commonly used for RNA degradation assessment. After treating cells with 5 μg/ml actinomycin D, we observed faster *PTEN* degradation in shYTHDC1 cells when compared with that in shNC cells (Figure 1J,K). The half‐lives of *PTEN* were 5.88 ± 0.41, 5.66 ± 1.09 and 9.55 ± 0.75 h in T24 shYTHDC1‐1, shYTHDC1‐2 and shNC cells, respectively, and 3.86 ± 0.41, 3.96 ± 0.45 and 6.34 ± 0.42 h in U3 shYTHDC1‐1, shYTHDC1‐2 and shNC cells, respectively. These results indicate that silencing YTHDC1 destabilizes PTEN mRNA. Different locations of PTEN suggest different roles, whereas cytoplasmic PTEN is specifically responsible for PI3K phosphorylation and AKT activation.^9^, ^12^ In an immunofluorescence (IF) assay, it was found that PTEN expressed both in the cytoplasm and nuclear, whereas p‐AKT (ser473) particularly located in the cytoplasm (Figure 1L). Moreover, we did not observe any difference in PTEN location between shNC and shYTHDC1 cells. Therefore, silencing YTHDC1 reduces PTEN expression by decreasing the stability of PTEN mRNA.

### 3.2 Lower YTHDC1 level indicates poor cisplatin response in bladder cancer

**FIGURE 2:**
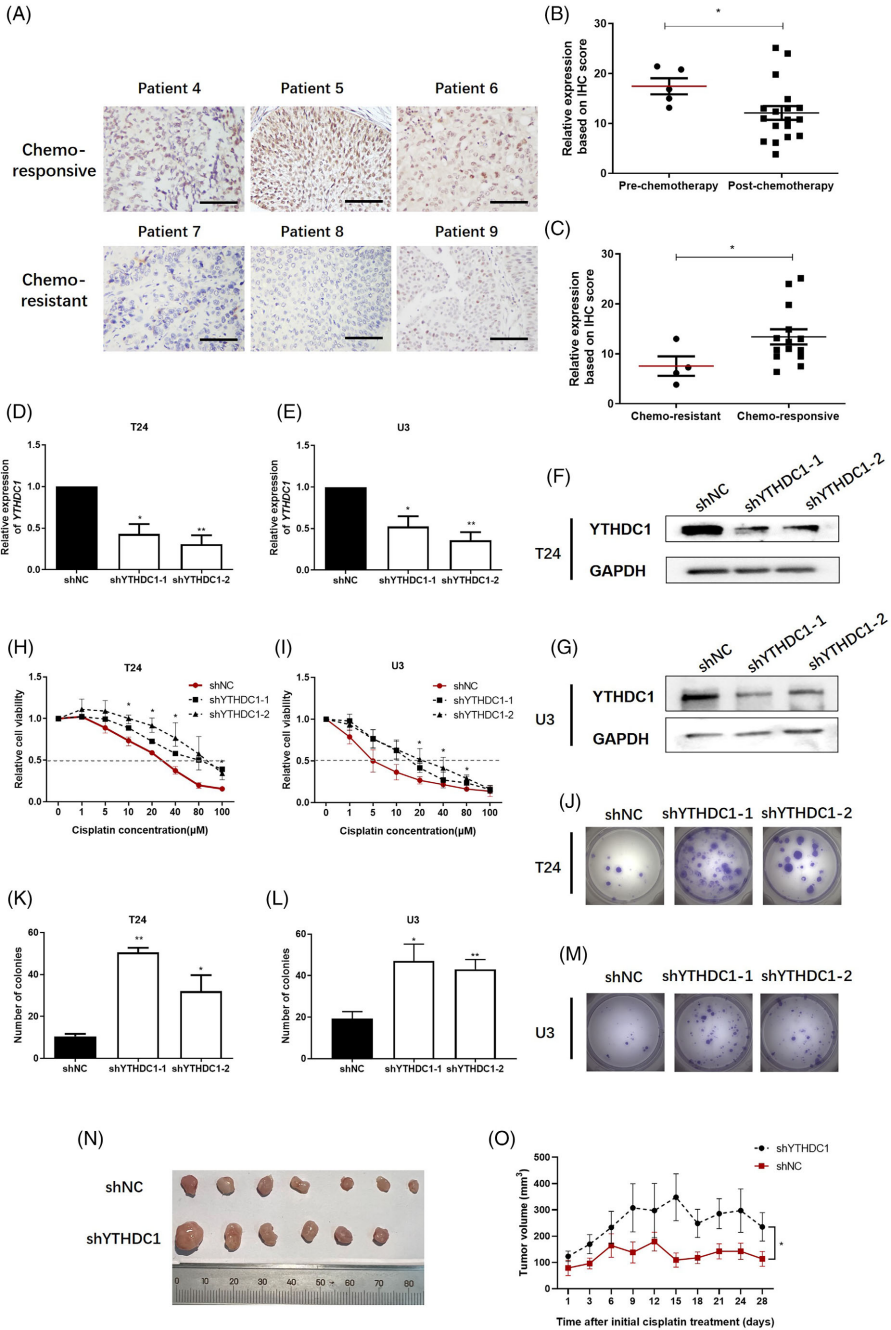
Lower YTHDC1 level indicates poor cisplatin response in bladder cancer. YTHDC1 level was detected by immunohistochemistry (IHC) assay in tumour sections from 20 bladder cancer patients that have received cisplatin‐containing chemotherapy. (A) Representative images show YTHDC1 expression in six identical patients. (B and C) Scatter plots show IHC scores for YTHDC1 staining. The scale bar indicates 50 μm. (D and E) Real‐time polymerase chain reaction (PCR) evaluated the expression of YTHDC1 on mRNA level. (F and G) The protein level of YTHDC1 was detected by Western blot. GAPDH was applied as an internal control both for quantitative real‐time PCR and Western blot detection. Data are presented as the mean ± SEM, and experiments were performed at least three times. (H and I) After 48 h of treatment with different doses of cisplatin, cell viabilities were measured by using Cell Counting Kit‐8 assay. Data are presented as the mean ± SEM, and experiments were performed at least three times. (J–M) After treatment with 20 μM cisplatin, the growth of single cells was measured after 2 weeks by a colony formation assay. Representative images are displayed. (K and L) Colonies with over 50 cells were counted. Data are presented as the mean ± SEM, experiments were performed at least three times. Mice that bearing bladder carcinoma xenograft were treated with cisplatin (3 mg/kg) per week. The response to cisplatin treatment was reflected by the change of tumour size. (N) Representative images illustrate dissected tumour samples at the end of experiment. (O) The size of tumour xenografts was measured every 3 days and tumour growth curve was displayed. shYTHDC1‐2 cells were selected and used to construct YTHDC1 silencing xenografts. Data are presented as the mean ± SEM.

We hypothesized that YTHDC1 might regulate PTEN expression to exert cisplatin resistance. Hence, we analysed IHC scores with YTHDC1 staining among 20 bladder cancer patients that have received cisplatin‐containing chemotherapy. Patients were divided into chemo‐responsive and chemo‐resistant groups, according to patients' outcome that was evaluated after receiving standard chemotherapy. As illustrated, the expression of YTHDC1 decreased after chemotherapy (Figure 2B), and lower YTHDC1 level was significantly observed in chemo‐resistant samples (Figure 2A,C).

Next, we constructed T24 and U3 bladder cancer cells with stably YTHDC1 silencing lenti‐virus, and constructed T24 bladder cancer cells with stably YTHDC1 over‐expressing lenti‐virus to evaluate the effect of YTHDC1 on cisplatin resistance. The silenced cells were named as shYTHDC1‐1 and shYTHDC1‐2, whereas shNC was used as a control. The cell that over‐expresses YTHDC1 was named as YTHDC1, whereas Vector was used as a control. These transfected cells were evaluated by quantitative real‐time PCR and Western blot to confirm YTHDC1 level, which are shown in Figures 2D,F and S2A,B for T24 cells and Figure 2E,G for U3 cells.

Both two cell lines were first treated with cisplatin for 4 h to induce DNA damage. It was found that, after 48 h of cisplatin treatment, cell viability was enhanced in YTHDC1 silencing bladder cancer cells, as demonstrated with IC50 for cisplatin, which increased from 46.18 ± 0.63 μM in shNC cells to 78.38 ± 13.32 μM in shYTHDC1‐1 and 85.98 ± 12.35 μM in shYTHDC1‐2 T24 cells (Figure 2H), and increased from 19.42 ± 5.11 μM in shNC cells to 42.30 ± 4.85 μM in shYTHDC1‐1 and 43.78 ± 4.03 μM in shYTHDC1‐2 U3 cells (Figure 2I). Oppositely, cell viability was reduced in YTHDC1 over‐expressing bladder cancer cells, demonstrated with IC50 for cisplatin reduced from 38.31 ± 7.63 μM in Vector cells to 6.38 ± 5.49 μM in YTHDC1 over‐expressing T24 cells (Figure S2C). Besides, in a colony formation assay, more colonies were counted in the shYTHDC1 group (Figure 2J–M) compared with that in the shNC cells, and less colonies were counted in the YTHDC1 over‐expressing cells (Figure S2D,E) compared with that in the Vector cells.

Based on the in vitro results demonstrated above, we next examined the effect of YTHDC1 on cisplatin resistance in bladder carcinoma xenograft. All mice with bladder carcinoma xenograft were treated with cisplatin(3 mg/kg) per week. At the end of the fourth week of cisplatin treatment, the surviving mice were sacrificed and the tumour xenografts were dissected. As demonstrated, we observed larger tumour size (Figure 2N) and quicker tumour growth (Figure 2O) in the shYTHDC1 cells when compared with that in the shNC cells, and smaller tumour size (Figure S2F) and slower tumour growth (Figure S2G) in the YTHDC1 over‐expressing cells when compared with that in Vector cells. These results suggest that the shYTHDC1 xenografts are more resistant to cisplatin treatment than the shNC ones, and the YTHDC1 over‐expressing xenografts are more sensitive to cisplatin treatment than the Vector ones.

Taken together, these results indicate that YTHDC1 plays a critical role in cisplatin resistance in bladder cancer.

### 3.3 YTHDC1 level is positively associated with DNA damage degree in bladder cancer

**FIGURE 3:**
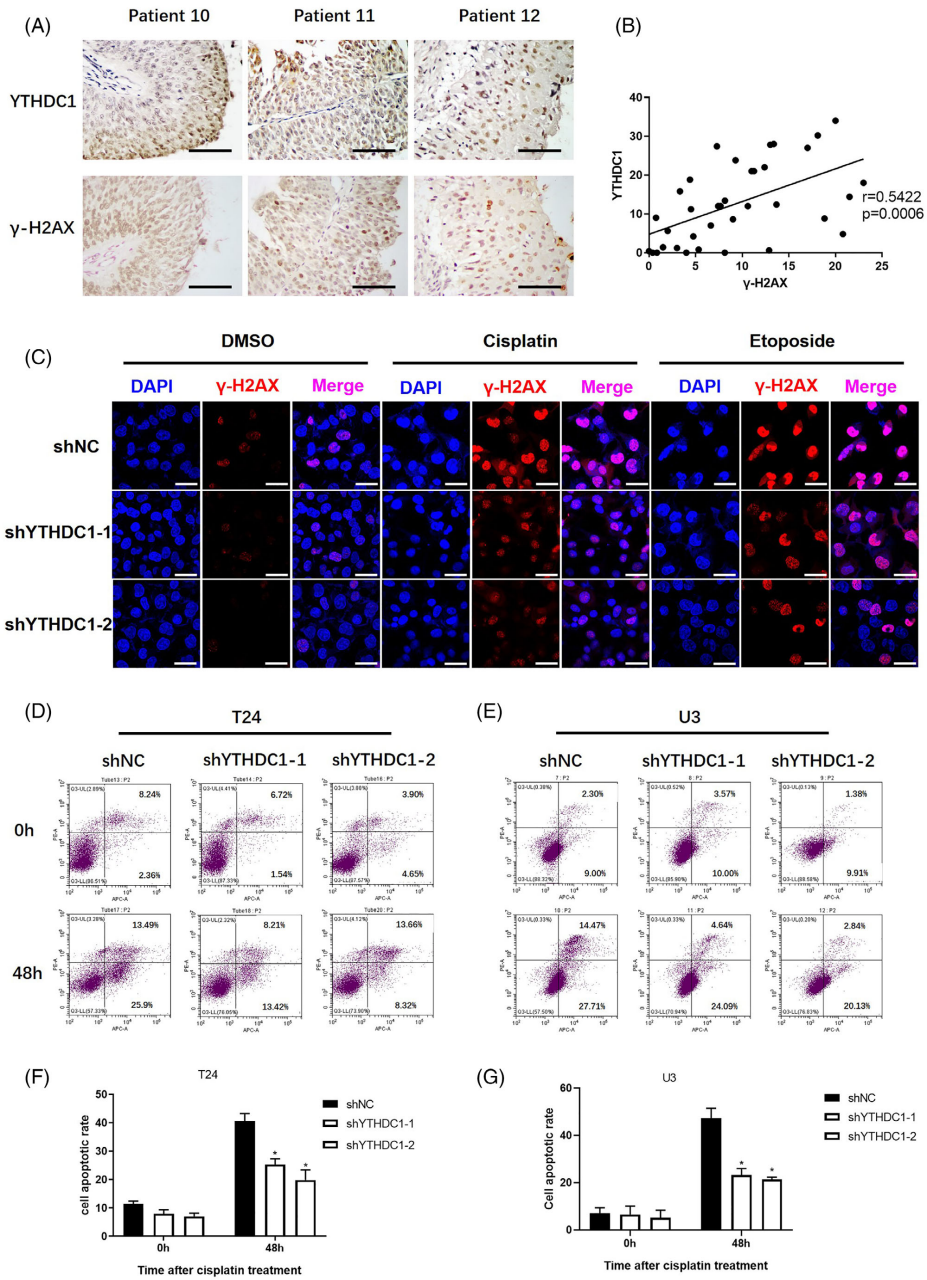
Lower expression of YTHDC1 alleviates DNA damage and apoptosis in bladder cancer. The YTHDC1 and γ‐H2AX levels were detected by an immunohistochemistry assay in tumour sections from 36 bladder cancer patients. The correlation between YTHDC1 and γ‐H2AX levels were analysed by using Graphpad Prism 8.0 software. (A) Representative images show YTHDC1 and γ‐H2AX expression levels in three identical patients. The scale bar indicates 50 μm. (B) Scatter plots show the correlation between YTHDC1 and γ‐H2AX level. Bladder cancer cells were treated with 20 μM cisplatin and 10 μM etoposide for 4 h, and 48 h later, the cells were fixed. The expression of γ‐H2AX was detected by using an immunofluorescence assay. (C) Representative images show the level of γ‐H2AX in different T24 bladder cancer cells. The scale bar indicates 40 μm. Experiments were performed at least three times. Annexin V‐AF647/PI staining was performed to evaluate the effect of cisplatin on cell apoptosis. Cells were treated with 40 μM cisplatin for 4 h and tested 48 h later. Cell positivity for Annexin V‐AF647/PI was detected by flow cytometry (D and E). The total apoptotic rate includes the sum of early and late apoptotic cells, which are shown in (F) and (G). Data are presented as the mean ± SEM, and experiments were performed at least three times.

Cisplatin conventionally induces DNA damage in cells.^4^ A reduced degree of DNA damage results in cell survival and leads to cisplatin resistance.^5^ We have demonstrated that YTHDC1 is a critical factor in cisplatin resistance in bladder cancer. We next tested the degree of DNA damage in bladder tumour sections with different YTHDC1 expression, and cells that were treated with cisplatin. In a panel of 36 sections with bladder cancer, we found a significant positive correlation between levels of YTHDC1 and γ‐H2AX, as illustrated by higher YTHDC1 expressing tumours displayed with higher γ‐H2AX expression (Figure 3A,B). To further verify the correlation between YTHDC1 expression and cisplatin‐induced DNA damage, we treated shNC and shYTHDC1 bladder cancer cells with cisplatin. After 48 h of recovery, γ‐H2AX was detected and evaluated by an IF assay (Figure 3C). As suggested, lower γ‐H2AX was observed in shYTHDC1 cells compared with that in shNC cells. The phenomenon was similarly observed when etoposide was used as a positive control.^22^ Besides, the level of γ‐H2AX was additionally detected by Western blot after cisplatin treatment (Figure 5B). It was found that lower YTHDC1 expression in cells was associated with lower γ‐H2AX expression. Above all, the results indicate that lower expression of YTHDC1 reduces the degree of cellular DNA damage in bladder cancer.

### 3.4 Lower expression of YTHDC1 affects cisplatin‐induced DDR in bladder cancer

The DDR includes a series of signal transductions events that involve cell apoptosis, cell cycle checkpoints and DNA repair. These events decide cell fate, while any abnormalities determine whether cells become sensitive or resistant to DNA damage‐inducing agents.^23^

Accumulated DNA damage leads to cell death most likely by inducing cell apoptosis.^5^ Hence, Annexin V‐AF647/PI staining was performed to evaluate the effects of YTHDC1 on cisplatin‐induced apoptosis and cell death events. Both shYTHDC1 and shNC cells were treated with 40 μM cisplatin for 4 h and then cultured for another 48 h. Cells were harvested and stained. Cell positivity for Annexin V‐AF647/PI was detected by flow cytometry. As shown in Figure 3D–G, after cisplatin treatment, the percentage of Annexin V‐AF647/PI positive cells was significantly lower in shYTHDC1 cells than that in shNC cells (Figure 3D–G). These results suggest that reduced YTHDC1 expression resists cisplatin‐induced apoptosis in bladder cancer.

**FIGURE 4:**
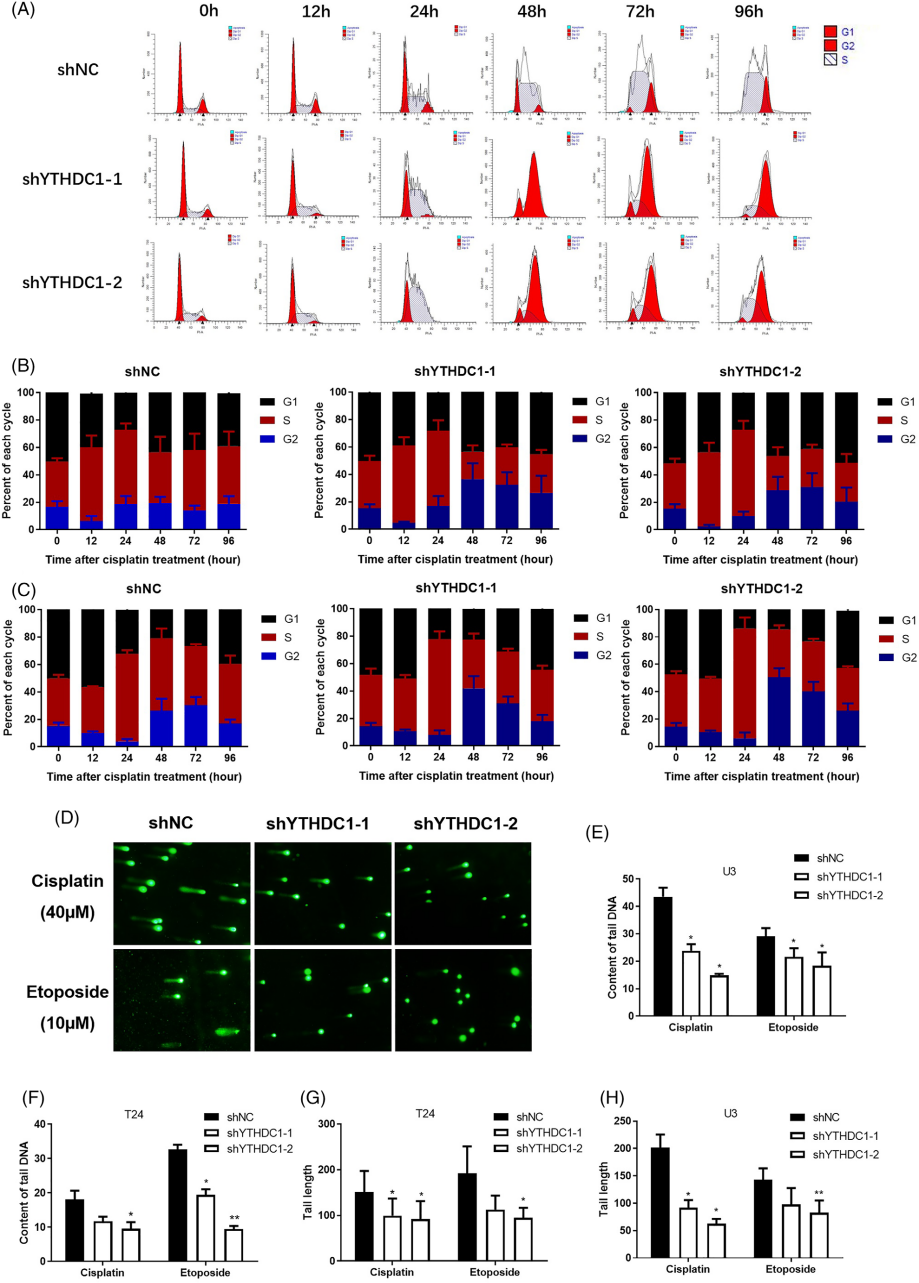
Lower expression of YTHDC1 affects cisplatin‐induced DNA damage response in bladder cancer. Cell aliquots were collected at 24‐h intervals after treatment with 20 μM cisplatin. The cell cycle distribution was analysed by flow cytometry. (A) Representative images show G1, S and G2 populations in T24 bladder cancer cells. The grouped histogram displays the dynamic mobility of the cell cycle distribution after cisplatin treatment in (B) T24 bladder cancer cells and (C) U3 bladder cancer cells. Data are presented as the mean ± SEM, and experiments were performed at least three times. For the comet assay, alkaline single‐cell electrophoresis was conducted 48 h after cisplatin or etoposide treatment, and the results were observed and photographed via microscopy. (D) Representative images show cell DNA fragments after cisplatin or etoposide treatment in T24 bladder cancer cells. Tail DNA content (E and F) and tail length (G and H) were calculated by using a CometScore 2.0 software. Data are presented as the mean ± SEM, and experiments were performed at least three times.

Following DNA damage, the cell cycle is firstly arrested, which provides cells with enough time to react. Cisplatin commonly induces DNA crosslinks, which typically leads to stalled replication forks, as well as S and G2/M arrest.^4^, ^5^, ^23^ To assess the role of YTHDC1 defect in these cell cycle events, we treated shYTHDC1 and shNC cells with 20 μM cisplatin for 4 h. Cell aliquots were collected for cell cycle analysis at 24‐h intervals. Consistent with many researches, we observed a predominant S and G2/M phase arrest in both cell lines. However, the exact mode was quite different when YTHDC1 expression was modulated. As illustrated, shYTHDC1 cells presented longer S phase and less G2/M arrest within 24 h after DNA damage introduction (Figure 4A–C). This is consistent with previous reports showing that impaired S phase arrest and prolonged G2/M arrest increase sensitivity to DNA‐damaging agents.^23^, ^24^, ^25^ After 48 h of cisplatin treatment, shYTHDC1 cells displayed with less cell number in S phase and more arrest in G2 phase (Figure 4A–C). Cell halts replication after DNA damage and initiates DNA repair.^26^, ^27^ Attenuated DNA damage impairs S phase replication stress, which supports our results and explains the cisplatin resistance of shYTHDC1 cells.^28^, ^29^

Efficient DNA repair could reduce the degree of DNA damage.^30^ To compare DNA repair capabilities between shYTHDC1 and shNC cells, a comet assay was applied. In the comet assay, greater DNA repair capacity should lead to less tail DNA content and tail monument. In contrast, if DNA repair is impaired, more tail DNA and a longer tail monument would be seen. After 48 h nap when receiving cisplatin, shYTHDC1 cells were observed to have shorter tail length and less tail DNA content when compared with that in shNC cells (Figure 4D–H). Similarly, a shorter tail monument was observed in shYTHDC1 cells when applying etoposide (Figure 4D–H). These comet assay results indicate that silencing YTHDC1 enhances DNA damage repair.

### 3.5 Activation of PI3K/AKT is responsible for YTHDC1 regulated DDR after cisplatin treatment in bladder cancer

**FIGURE 5:**
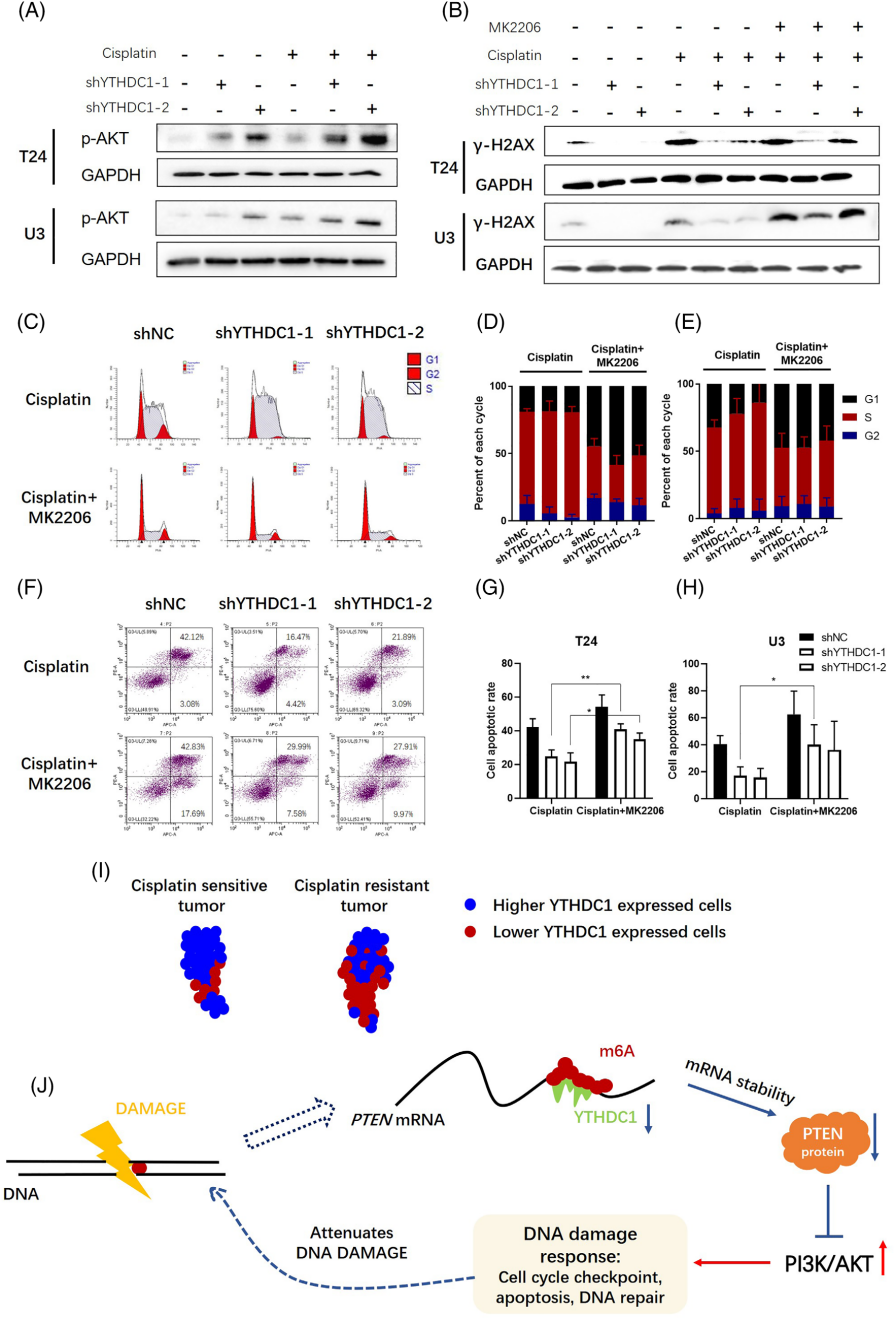
Activation of PI3K/AKT signalling is responsible for YTHDC1 regulated DNA damage response (DDR) after cisplatin treatment in bladder cancer. Bladder cancer cells were treated with 20 μM cisplatin for 4 h. (A) After 48 h, cells were collected and protein was extracted. The expression of p‐AKT(ser473) was examined by using Western blot assay. GAPDH was applied as an internal control, and experiments were performed at least three times. (B) To test the effect of MK2206 on the YTHDC1 associated DDR, cells were first treated with 20 μM cisplatin for 4 h, and then, the culture medium was changed to complete medium containing 10 μM MK2206. The expression of γ‐H2AX was examined by Western blot assay. GAPDH was applied as an internal control, and the experiments were performed at least three times. (C) Cell aliquots were collected 24 h after treatment with 20 μM cisplatin and 10 μM MK2206. The cell cycle distribution was analysed by flow cytometry. (C) Representative images show cell cycle distributions in T24 bladder cancer cells. The grouped histogram displays the dynamic mobility of the cell cycle distribution after treatment in (D) T24 bladder cancer cells and (E) U3 bladder cancer cells. Data are presented as the mean ± SEM, and experiments were performed at least three times. (F) Annexin V‐AF647/PI staining was performed to evaluate the synergistic effects of MK2206 on cisplatin‐induced cell apoptosis. Cells were treated with 40 μM cisplatin for 4 h and then 10 μM MK2206 for 48 h. Cell positivity for Annexin V‐AF647/PI was detected by flow cytometry. (F) Representative images show apoptosis in T24 bladder cancer cells. (G and H) The total apoptotic rate includes the sum of early and late apoptotic cells. Data are presented as the mean ± SEM, and experiments were performed at least three times. (I) and (J) present graphic abstract of this research. As illustrated, low YTHDC1 expression in cancer cells indicates poor cisplatin therapy outcome in bladder cancer patients, and low YTHDC1 expression results in destabilization of PTEN mRNA, which activates the AKT‐associated DDR and attenuates cisplatin‐induced DNA damage.

Repressing PI3K/AKT signalling is the classical function of PTEN.^9^, ^12^ By analysing sequencing data between shNC and shYTHDC1 bladder cancer cells, the signalling pathway of PI3K/AKT was significantly enriched (Figure S1B; Table S2). The activation of PI3K/AKT was also observed when processing bladder cancer cells with Western blot (Figure 5A) and IF assay (Figure 1L). As demonstrated, silencing YTHDC1 reduced PTEN expression, whereas the expression of p‐AKT (ser473) was increased. This effect was remarkably obvious when treating bladder cancer cells with cisplatin (Figure 5A). Taken together, silencing YTHDC1 reduces PTEN expression that in turn activates AKT signalling.

Accumulating evidence has implied that AKT participates in DDR.^11^ To test whether shYTHDC1 affects cisplatin‐induced drug resistance and DDR through activating AKT signalling, we adopted MK2206, a chemical compound to inhibit the allosteric regulation of AKT.^31^ By observing the indices of the DDR, it was found that MK2206 displayed a synergistic effect with cisplatin in bladder cancer cells. Treating shYTHDC1 cells with MK2206 increased cisplatin sensitivity and cell death (Figure 5F–H) when compared with those effects in shYTHDC1 cells treated only with cisplatin. However, MK2206 did not completely reverse the decreased cell apoptosis in shYTHDC1 cells when compared with that in shNC cells. These results suggest that YTHDC1 affects cisplatin resistance through mechanisms beyond activating AKT signalling.

In addition, cell cycle distribution and the DNA damage were also evaluated. After treating bladder cancer cells with cisplatin and MK2206 together, S phase replication arrest was reduced compared with cells that were treated with cisplatin alone, whereas the cell population in G2 phase and G1 phase was enlarged (Figure 5C–E). Interestingly, the synergistic effect of MK2206 was much stronger in shYTHDC1 cells compared with that in shNC cells. Therefore, we conclude that the shYTHDC1 regulated cell cycle checkpoint is at great extent induced by activating AKT signalling in bladder cancer. γ‐H2AX expression indicates the degree of DNA damage^30^ and was evaluated by Western blot. As illustrated, MK2206 increased the cisplatin‐induced γ‐H2AX expression (Figure 5B). However, the level of γ‐H2AX in shNC cells was stronger than that in shYTHDC1 cells. These results suggest that there might be other possible mechanisms for YTHDC1 in affecting cisplatin‐induced DNA damage repair, although AKT signalling participates in some of it.

### 3.6 The clinical relevance of YTHDC1 in bladder cancer

According to calculations from www.kmplot.com, *YTHDC1* displays the greatest prognostic value among the 21 tumour types involved, based on TCGA datasets (Figure S3A). As suggested, lower *YTHDC1* expression in bladder cancer indicated worse overall survival (Figure S3B). Besides, in 33 bladder cancer samples we collected, similar result that lower YTHDC1 indicated worse overall survival was identified (Figure S4C; Table S1). Further separating tumour samples in TCGA datasets by tumour mutation burden (TMB), it appeared that lower *YTHDC1* expression coupled with lower TMB indicated worse overall survival (Figure S3C), as well as the same results were found in patients once lower *YTHDC1* expression was coupled with lower *TP53* level (Figure S4A,B). These results indicate a possible tumour suppressive role of YTHDC1.

Among the different pathology of bladder cancer in TCGA datasets and 77 bladder cancer samples we collected, YTHDC1 strictly expressed in the T1 stage, whereas its level reduced when the disease progressed to or beyond the T2 stage (Figures S3D and S4D; Table S1). The discrepancy in *YTHDC1* expression was also observed in different molecular subtypes of bladder cancer and higher *YTHDC1* level was identified in the neuronal subtype (Figure S3E).

In a cohort of patients with 51 tumour sections and 37 sections of adjacent normal tissues, low YTHDC1 level was detected in malignant tumour by IHC assay (Figure S3G,H), as well as in 16 paired tumour/peri‐tumour samples by quantitative real‐time PCR (Figure S3I). This observation was similarly verified when analysing TCGA datasets (Figure S3F).

In a panel of *in vitro* cell lines, including the normal cell line: SV‐HUC‐1, and the bladder cancer cell lines: T24, U3, 5637, J82 and RT4, higher level of YTHDC1 was observed in SV‐HUC‐1 cells compared with that in bladder cancer cells, whereas in bladder cancer cells, varied expression of YTHDC1 was observed (Figure S3J,K).

## 4 DISCUSSION

Resisting cisplatin‐induced DNA damage significantly contributes to cancer drug resistance.^5^, ^30^ Abnormal DDR in cancer promotes DNA repair or makes cells more prone to DNA damage.^29^, ^32^ In addition to frequent mutations in genes involved in the DDR, irregular activation of oncogenes and repression of tumour suppressors are other upstream regulators of aberrant DDR in cancer.^30^, ^33^ PTEN loss and activation of AKT signalling is a classic example.^11^, ^12^ It was found that lack of PTEN expression improved DNA repair ability and apoptosis evasion.^7^, ^10^ This role was further confirmed by a low PTEN level was observed in chemo‐resistant cancer patients.^34^ Although PTEN plays an ultimate role in caner, clinical therapeutic approaches that focused on the PTEN/AKT axis did not achieve expected outcome.^35^ On the one hand, AKT signalling is one of the most frequently mutated pathways in cancer. However, the current pharmaceutical options cannot effectively cover the target in most patients.^36^ On the other hand, PTEN widely participates in cell behaviours.^37^ Direct targeting of PTEN will of no doubt lead to inestimable side effects. Hence, further research on how PTEN is regulated, would help uncover more clues to identify better tumour specific‐targets and biomarkers.

In this study, we showed that YTHDC1 regulated PTEN/PI3K/AKT signalling pathway in an m^6^A‐dependent manner to affect chemotherapy efficacy. Silencing YTHDC1 reduced PTEN expression but activated PI3K/AKT signalling by destabilizing PTEN mRNA. Reducing YTHDC1 expression promoted drug resistance to cisplatin, and activated the DDR, which includes quicker cell cycle recovery, apoptosis evasion and enhanced capacity for DNA repair, whereas these effects were attenuated when the MK2206, a PI3K/AKT inhibitor was applied. Moreover, in a cohort of bladder cancer patients, the expression of YTHDC1 was positively correlated with PTEN and γ‐H2AX levels, and lower YTHDC1 levels were specifically found in chemo‐resistant patients. The above findings suggest that YTHDC1 is a critical factor affecting intrinsic cisplatin resistance in bladder cancer, particularly by regulating the PTEN/AKT/DDR pathway.

Furthermore, we also found a reduced YTHDC1 expression in bladder cancer patients who have received chemotherapy, as well as in bladder cancer cells. Accordingly, reduced YTHDC1 expression was found to be consistent with decreased PTEN levels and p‐AKT upregulation after cisplatin treatment. Reduced PTEN levels after chemotherapy in cancer have been previously demonstrated to participate in acquired chemoresistance.^34^ These findings suggest that YTHDC1 might play a role in acquired drug resistance in bladder cancer.

Although we demonstrated that reducing YTHDC1 activated the DDR via PTEN repression and AKT activation, impairing AKT function did not fully rescue the enhanced cisplatin resistance and the DDR induced by YTHDC1 silencing. This discrepancy suggests additional mechanisms by which YTHDC1 regulates cisplatin resistance. In addition to the DDR, drug efflux and cell autophagy are the other mechanisms that contribute to cisplatin resistance in cancers.^5^, ^23^ After bladder cancer cells were treated with cisplatin, we did not find temporal/longitudinal difference in the γ‐H2AX increase between shNC and shYTHDC1 cells. This suggests that YTHDC1 is less likely to mediate cisplatin resistance by affecting drug efflux. Fortunately, treatment of bladder cancer cells with shYTHDC1 suppressed cisplatin‐induced cell autophagy (data not shown). Cisplatin stimulates autophagy for cell survival at first.^38^ However, over‐activation of autophagy exacerbates cisplatin sensitivity.^38^ PI3K/AKT signalling typically represses autophagy for cell protection.^39^, ^40^ Hence, the suppressed autophagy supports the previously mentioned AKT activation that was observed when YTHDC1 was silenced. But how does autophagy participate in shYTHDC1‐mediated cisplatin resistance? Autophagy was reported to affect DNA repair via SQSTM1/P62, a core unit in driving autophagic flux.^41^ As investigated, SQSTM1 represses H2A ubiquitination, which prevents DNA repair factors from being recruited to damaged sites, whereas silencing of SQSTM1 promotes DNA repair.^41^, ^42^ Besides, YTHDC1 was reported to stabilize SQSTM1 nuclear mRNA in diabetic keratinocytes.^43^ This effect was consistent in bladder cancer cells (data not shown). Taken together, these results suggest that silencing YTHDC1 suppresses autophagy to promote cisplatin resistance and DNA repair via destabilizing *SQSTM1* in bladder cancer. Furthermore, autophagy was also reported to mediate protein metabolism, including DDR‐related factors.^38^, ^44^, ^45^, ^46^, ^47^ Hence, the enhanced DDR in shYTHDC1 bladder cancer cells might be explained by reduced autophagic protein degradation.

YTHDC1 expresses at significantly lower levels in malignant tissues. Compared with advanced tumours, YTHDC1 expresses at higher levels in the T1 stage. These discoveries suggest that YTHDC1 might play a tumour suppressive role in early carcinogenesis. We have shown that YTHDC1 mediates the DDR. Survival under constant DNA damage is the most critical hallmark of cancer formation.^48^ Enhanced DNA repair in cells with lower YTHDC1 expression makes cells more prone to tolerate DNA damage attack, thus creating suitable conditions for mutation‐mediated oncogenesis.^49^ However, in cells with higher YTHDC1 expression, severe DNA damage directly leads to the cell death, which can prevent malignant transformation. Furthermore, the above idea is supported by our findings in senescence studies between shNC and shYTHDC1 cells. Cell senescence represents ageing, which is the result of accumulated DNA damage and genomic instability.^50^, ^51^ We found obviously lower β‐galactosidase staining in shYTHDC1 cells than that in shNC cells (data not shown). Therefore, the above evidence suggests that low YTHDC1 expression is a DNA damage‐prone feature that can promote tumorigenesis.

In summary, we uncovered a novel mechanism that explains how PTEN is regulated in bladder cancer. We also demonstrated for the first time that YTHDC1 is a critical factor in cisplatin resistance in bladder cancer. Low expression of YTHDC1 indicates cisplatin resistance in bladder cancer patients and cells. Silencing YTHDC1 enables to activate the DDR through PTEN loss‐mediated AKT activation. These features suggest that YTHDC1 could be used as a potential biomarker to stratify patients who will be sensitive to cisplatin‐based chemotherapy. Treating patients with lower YTHDC1 expression levels with cisplatin combined with MK2206 could be a novel strategy to mitigate cisplatin resistance in bladder cancer.

## AUTHOR CONTRIBUTIONS

Dr Yinjie Su originally designed the project, performed all experiments and wrote the manuscript. Dr Tianxin Lin and Dr Bo Wang conceptualized the research and supervised the study. Dr Jian Huang and Dr Ming Huang helped collect clinical bladder cancer tissue samples. All authors revised and edited the manuscript.

## CONFLICT OF INTEREST

The authors declare no conflict of interest.

## Supporting information


**TABLE S1.** The clinical and pathological data of patients with bladder cancer.

**TABLE S2.** The sequencing data between shNC and shYTHDC1 T24 bladder cancer cells.

**FIGURE S1.** Silencing YTHDC1 down‐regulates PTEN but activates PI3K/AKT signalling in human bladder cancer.
A: The correlation between *YTHDC1* and *PTEN* expression levels was analysed with TCGA datasets. Plot was downloaded from gepia.cancer‐pku.cn. B: Graphs show top 15 enriched pathways between shNC and shYTHDC1 T24 bladder cancer cells.
**FIGURE S2.** Over‐expressing YTHDC1 promotes cisplatin sensitivity in bladder cancer cells.
A: Real‐time PCR evaluated the expression of YTHDC1 on mRNA level. B: The protein level of YTHDC1 was detected by Western blot. GAPDH was applied as an internal control both for quantitative real‐time PCR and Western blot detection. Data are presented as the mean ± SEM, and experiments were performed at least three times. C: After 48 h of treatment with different doses of cisplatin, cell viabilities were measured by using CCK8 assay. Data are presented as the mean ± SEM, and experiments were performed at least three times. D: After receiving 20 μM cisplatin treatment, the growth of single cells was measured after 2 weeks by a colony formation assay. Representative images are displayed. E: Colonies with over 50 cells were counted. Data are presented as the mean ± SEM, and experiments were performed at least three times. Mice that bearing bladder carcinoma xenograft were treated with cisplatin (3 mg/kg) per week. The response to cisplatin treatment was reflected by change of tumour size. F: Representative images illustrate dissected tumour samples at the end of experiment. G: The size of tumour xenografts was measured every 3 days and tumour growth curve was displayed. Data are presented as the mean ± SEM.
**FIGURE S3.** Clinical values of YTHDC1 in human bladder cancer.
The clinical values of YTHDC1 expression in bladder cancers were analysed with TCGA datasets. A: The prognostic value of *YTHDC1* in 21 cancer types is presented. B: Bladder cancer patients were separated by *YTHDC1* expression, with 295 individuals in the high‐*YTHDC1* group and 109 individuals in the low‐*YTHDC1* group. Lower *YTHDC1* expression indicates poor survival of patients. C: Bladder cancer patients were further divided based on both the *YTHDC1* level and tumour mutation burden (TMB). Kaplan–Meier analysis with a log rank test displayed that lower *YTHDC1* coupled with lower TMB suggested worse survival of patients. D: Expression of *YTHDC1* in different pathological stages of bladder cancer is presented. The plot was downloaded from the UALCAN analysis page. E: The expression of *YTHDC*1 in different molecular subtypes of bladder cancer is presented. The plot was downloaded from the UALCAN analysis page. F: The expression of *YTHDC1* in normal tissue (n = 19) and bladder cancer tissues (n = 408) are presented, p < 0.01. The plot was downloaded from the UALCAN analysis page. Besides, the expression of YTHDC1 was also analysed in tissue samples we collected and in vitro cell lines. As illustrated, the YTHDC1 level in 51 tumour and 37 adjacent normal tissues was analysed by using an IHC assay. Scatter plots of YTHDC1 IHC scores are shown in G (p < 0.01), and the representative images are shown in H. The scale bar indicates 50 μm. I: YTHDC1 mRNA expression was detected by quantitative real‐time PCR in 16 paired of human bladder cancer tissues and adjacent normal controls (p < 0.01). The expression of GAPDH was used as an internal control. J: YTHDC1 mRNA expression was analysed quantitatively in a panel of cell lines. The expression of GAPDH was used as an internal control. Data are presented as the mean ± SEM, and experiments were performed at least three times. K: The protein levels of YTHDC1 were evaluated by Western blot in a panel of cell lines. The expression of GAPDH was used as an internal control, and experiments were performed at least three times.
**FIGURE S4.** Clinical values of YTHDC1 in human bladder cancer.
A and B: The bladder cancer patients in TCGA dataset were divided on both the *YTHDC1* and *TP53* levels together. Kaplan–Meier analysis with a log rank test displayed that lower *YTHDC1* expression coupled with lower *TP53* expression showed worse patient survival. C: 33 bladder cancer patients we collected were separated by YTHDC1 expression, with 16 individuals in the high‐YTHDC1 group and 17 individuals in the low‐YTHDC1 group. The median of YTHDC1 expression was taken as cut‐off. Kaplan–Meier analysis with a log rank test displayed that lower YTHDC1 expression indicated poor survival of patients. D: The expression of YTHDC1 in different pathological stages was analysed in 77 bladder cancer samples we collected.

## Data Availability

Original data in our study are available upon request.
