# Supplementary material for: YTHDC1 positively regulates PTEN expression and plays a critical role in cisplatin resistance of bladder cancer
Source: Cell Prolif. 2023 Apr 17;56(7):e13404. doi: 10.1111/cpr.13404 (PMC10334275; doi:10.1111/cpr.13404)
Supplement: Supplementary file 3 — FIGURE S1. Silencing YTHDC1 down‐regulates PTEN but activates PI3K/AKT signalling in human bladder cancer. A: The correlation between YTHDC1 and PTEN expression levels was analysed with TCGA datasets. Plot was downloaded from gepia.cancer‐pku.cn. B: Graphs show top 15 enriched pathways between shNC and shYTHDC1 T24 bladder cancer cells. FIGURE S2. Over‐expressing YTHDC1 promotes cisplatin sensitivity in bladder cancer cells. A: Real‐time PCR evaluated the expression of YTHDC1 on mRNA level. B: The protein level of YTHDC1 was detected by Western blot. GAPDH was applied as an internal control both for quantitative real‐time PCR and Western blot detection. Data are presented as the mean ± SEM, and experiments were performed at least three times. C: After 48 h of treatment with different doses of cisplatin, cell viabilities were measured by using CCK8 assay. Data are presented as the mean ± SEM, and experiments were performed at least three times. D: After receiving 20 μM cisplatin treatment, the growth of single cells was measured after 2 weeks by a colony formation assay. Representative images are displayed. E: Colonies with over 50 cells were counted. Data are presented as the mean ± SEM, and experiments were performed at least three times. Mice that bearing bladder carcinoma xenograft were treated with cisplatin (3 mg/kg) per week. The response to cisplatin treatment was reflected by change of tumour size. F: Representative images illustrate dissected tumour samples at the end of experiment. G: The size of tumour xenografts was measured every 3 days and tumour growth curve was displayed. Data are presented as the mean ± SEM. FIGURE S3. Clinical values of YTHDC1 in human bladder cancer. The clinical values of YTHDC1 expression in bladder cancers were analysed with TCGA datasets. A: The prognostic value of YTHDC1 in 21 cancer types is presented. B: Bladder cancer patients were separated by YTHDC1 expression, with 295 individuals in the high‐YTHDC1 group and 109 indivi [file CPR-56-e13404-s001.docx]

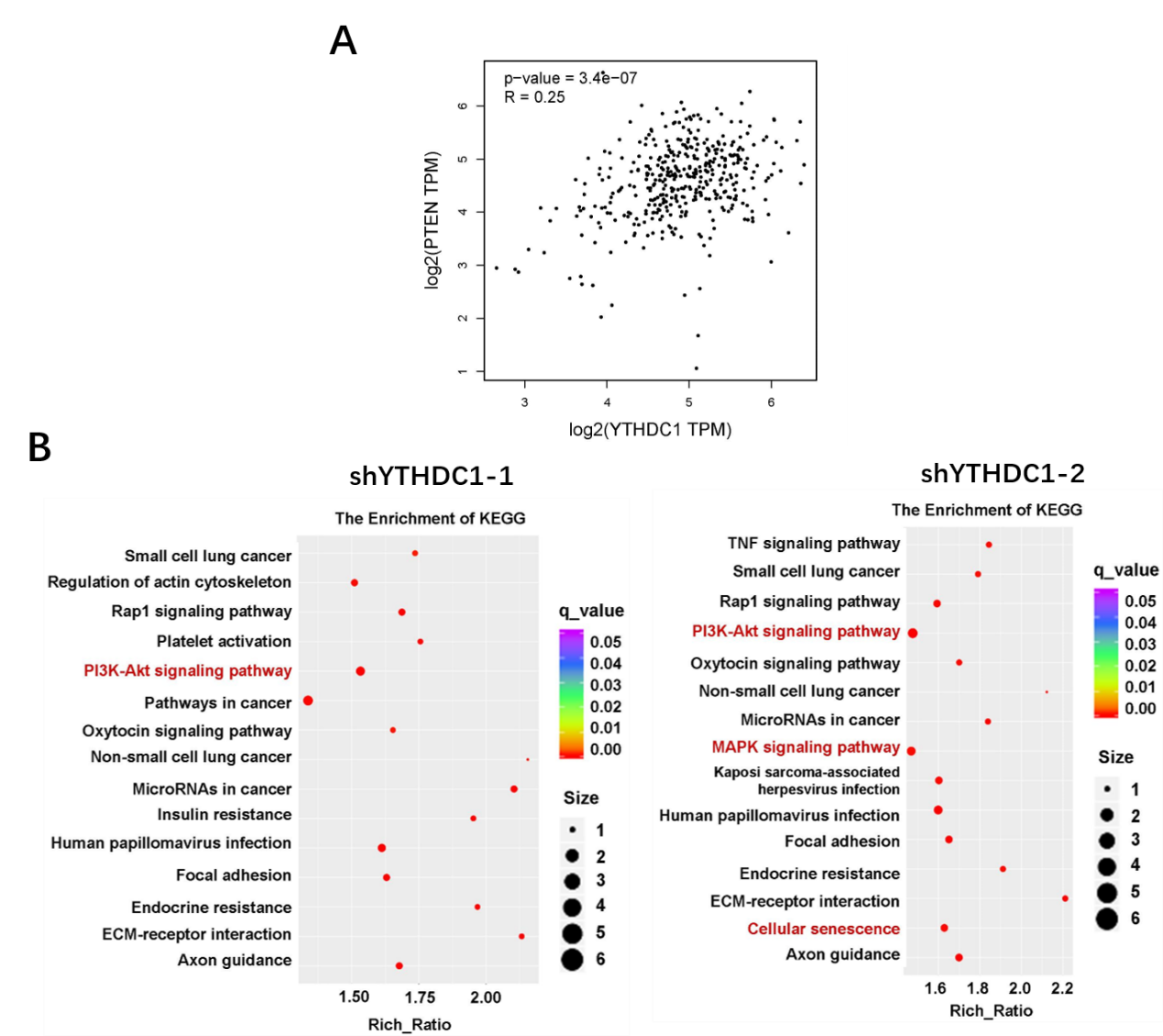


**Figure.S1 Silencing YTHDC1 down-regulates PTEN but activates PI3K/AKT signaling in human bladder cancer.**

A: The correlation between *YTHDC1* and *PTEN* expression levels was analysed with TCGA datasets. Plot was downloaded from gepia.cancer-pku.cn. B: Graphs show top 15 enriched pathways between shNC and shYTHDC1 T24 bladder cancer cells.


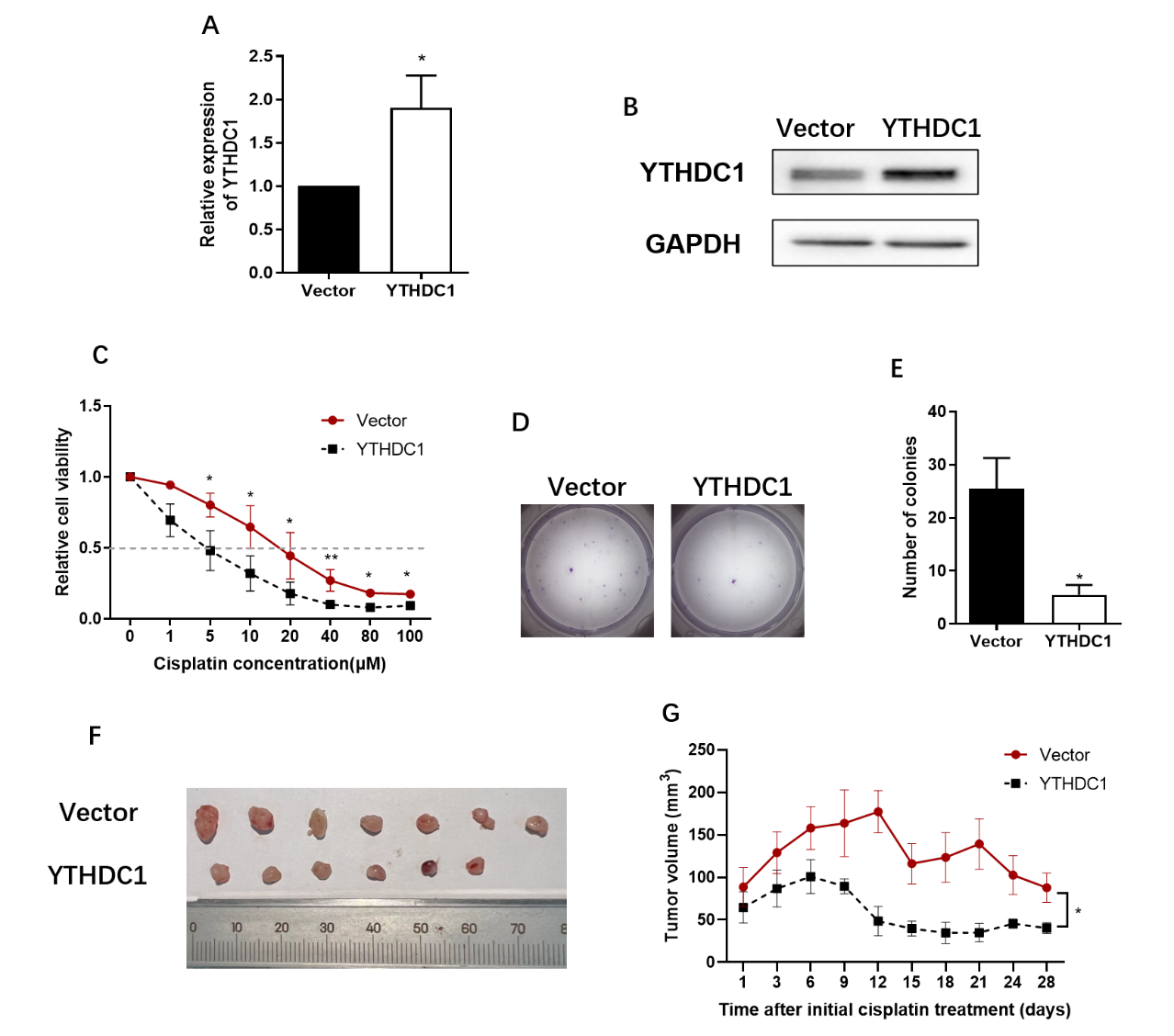


**Figure.S2 Over-expressing YTHDC1 promotes cisplatin sensitivity in bladder cancer cells.**

A: Real‐time PCR evaluated the expression of YTHDC1 on mRNA level. B: The protein level of YTHDC1 was detected by Western blot. GAPDH was applied as an internal control both for quantitative real‐time PCR and Western blot detection. Data are presented as the mean ± SEM, and experiments were performed at least three times. C: After 48 h of treatment with different doses of cisplatin, cell viabilities were measured by using CCK8 assay. Data are presented as the mean ± SEM, and experiments were performed at least three times. D: After receiving 20 μM cisplatin treatment, the growth of single cells was measured after 2 weeks by a colony formation assay. Representative images are displayed. E: Colonies with over 50 cells were counted. Data are presented as the mean ± SEM, and experiments were performed at least three times. Mice that bearing bladder carcinoma xenograft were treated with cisplatin (3 mg/kg) per week. The response to cisplatin treatment was reflected by change of tumour size. F: Representative images illustrate dissected tumour samples at the end of experiment. G: The size of tumour xenografts was measured every 3 days and tumour growth curve was displayed. Data are presented as the mean ± SEM.


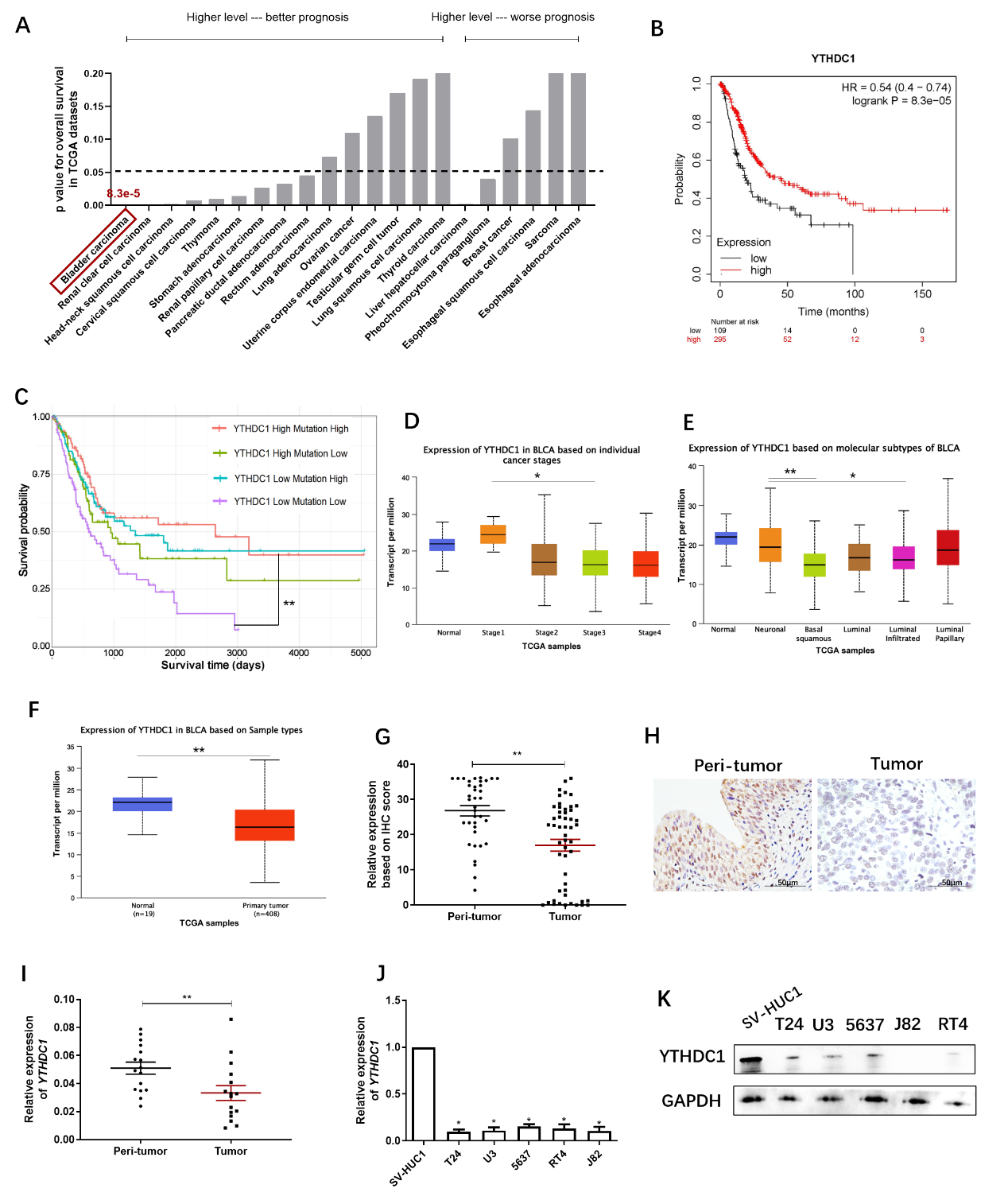


**Figure.S3 Clinical values of YTHDC1 in human bladder cancer.**

The clinical values of YTHDC1 expression in bladder cancers were analysed with TCGA datasets. A: The prognostic value of YTHDC1 in 21 cancer types is presented. B: Bladder cancer patients were separated by YTHDC1 expression, with 295 individuals in the high‐YTHDC1 group and 109 individuals in the low‐YTHDC1 group. Lower YTHDC1 expression indicates poor survival of patients. C: Bladder cancer patients were further divided based on both the YTHDC1 level and tumour mutation burden (TMB). Kaplan–Meier analysis with a log rank test displayed that lower YTHDC1 coupled with lower TMB suggested worse survival of patients. D: Expression of YTHDC1 in different pathological stages of bladder cancer is presented. The plot was downloaded from the UALCAN analysis page. E: The expression of YTHDC1 in different molecular subtypes of bladder cancer is presented. The plot was downloaded from the UALCAN analysis page. F: The expression of YTHDC1 in normal tissue (n = 19) and bladder cancer tissues (n = 408) are presented, p < 0.01. The plot was downloaded from the UALCAN analysis page. Besides, the expression of YTHDC1 was also analysed in tissue samples we collected and *in vitro* cell lines. As illustrated, the YTHDC1 level in 51 tumour and 37 adjacent normal tissues was analysed by using an IHC assay. Scatter plots of YTHDC1 IHC scores are shown in G (p < 0.01), and the representative images are shown in H. The scale bar indicates 50 μm. I: YTHDC1 mRNA expression was detected by quantitative real‐time PCR in 16 paired of human bladder cancer tissues and adjacent normal controls (p < 0.01). The expression of GAPDH was used as an internal control. J: YTHDC1 mRNA expression was analysed quantitatively in a panel of cell lines. The expression of GAPDH was used as an internal control. Data are presented as the mean ± SEM, and experiments were performed at least three times. K: The protein levels of YTHDC1 were evaluated by Western blot in a panel of cell lines. The expression of GAPDH was used as an internal control, and experiments were performed at least three times.


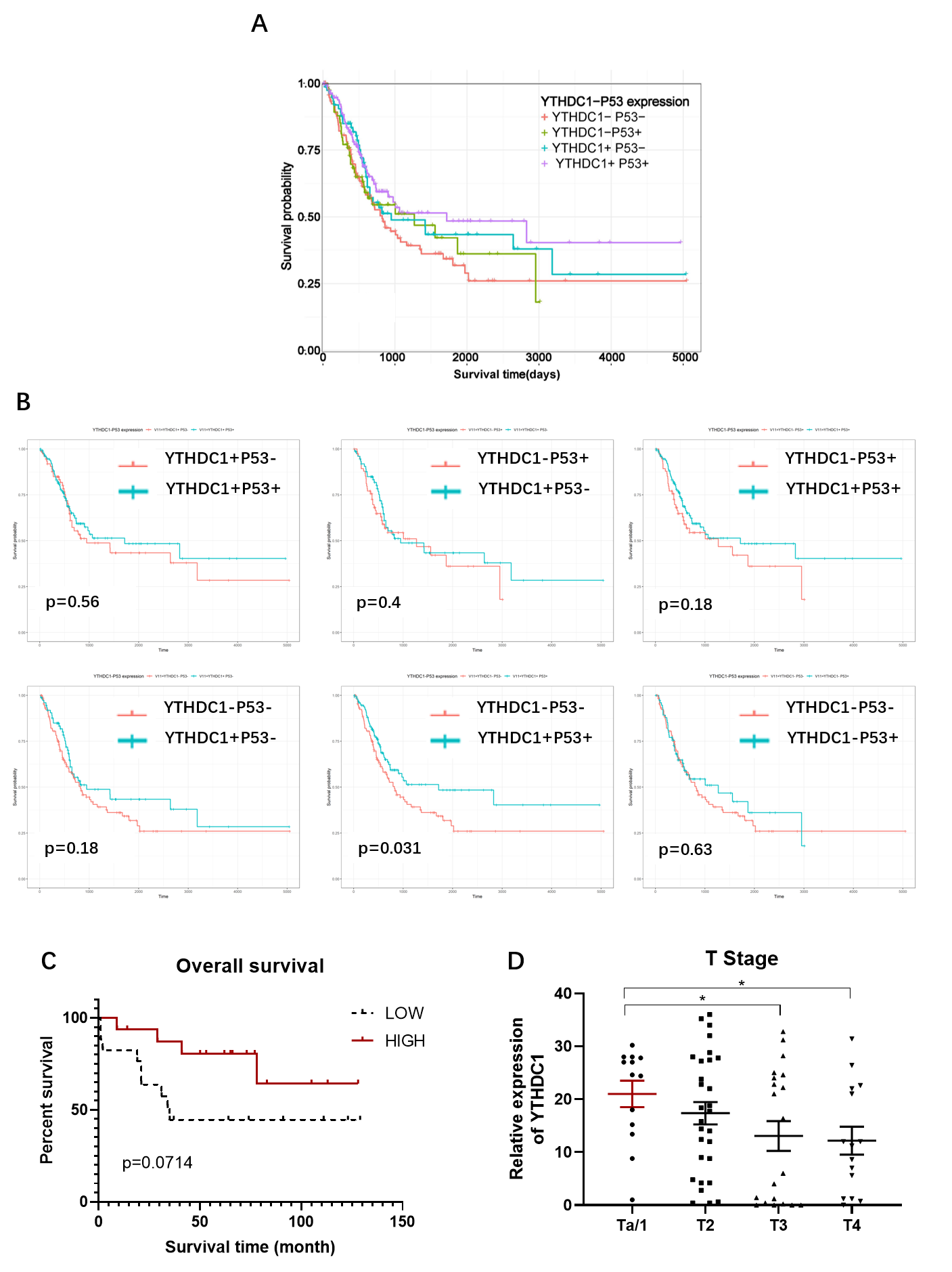


**Figure.S4 Clinical values of YTHDC1 in human bladder cancer.**

A and B: The bladder cancer patients in TCGA dataset were divided on both the *YTHDC1* and *TP53* levels together. Kaplan–Meier analysis with a log rank test displayed that lower *YTHDC1* expression coupled with lower *TP53* expression showed worse patient survival. C: 33 bladder cancer patients we collected were separated by YTHDC1 expression, with 16 individuals in the high‐YTHDC1 group and 17 individuals in the low‐YTHDC1 group. The median of YTHDC1 expression was taken as cut‐off. Kaplan–Meier analysis with a log rank test displayed that lower YTHDC1 expression indicated poor survival of patients. D: The expression of YTHDC1 in different pathological stages was analysed in 77 bladder cancer samples we collected.
